# Inferring differentially expressed pathways using kernel maximum mean discrepancy-based test

**DOI:** 10.1186/s12859-016-1046-1

**Published:** 2016-06-06

**Authors:** Esteban Vegas, Josep M. Oller, Ferran Reverter

**Affiliations:** Department of Statistics, University of Barcelona, Diagonal, 643, Barcelona, 08028 Spain; Center of Genomic Regulation, Parc de Recerca Biomedica de Barcelona, Dr. Aiguader, 88, Barcelona, 08003 Spain

**Keywords:** Kernel-based methods, Kernel maximum mean test, Omics data integration

## Abstract

**Background:**

Pathway expression is multivariate in nature. Thus, from a statistical perspective, to detect differentially expressed pathways between two conditions, methods for inferring differences between mean vectors need to be applied. Maximum mean discrepancy (MMD) is a statistical test to determine whether two samples are from the same distribution, its implementation being greatly simplified using the kernel method.

**Results:**

An MMD-based test successfully detected the differential expression between two conditions, specifically the expression of a set of genes involved in certain fatty acid metabolic pathways. Furthermore, we exploited the ability of the kernel method to integrate data and successfully added hepatic fatty acid levels to the test procedure.

**Conclusion:**

MMD is a non-parametric test that acquires several advantages when combined with the kernelization of data: 1) the number of variables can be greater than the sample size; 2) omics data can be integrated; 3) it can be applied not only to vectors, but to strings, sequences and other common structured data types arising in molecular biology.

## Background

A challenging topic for the bioinformatics community is how to combine data from multiple sources to increase biological knowledge. Integrating data from various different sources is not simply a matter of summing the results of each separate source; rather, it requires the analysis at the same time of all variables from various sources [1–3].

Nowadays, there are many methods to integrating heterogeneous data but kernel-based methods are usually the most powerful [4, 5]. Kernel-based methods have an extensive variety of kernels in which they can be used for each source of data. Thus, a first step to data integration is to choose an appropriate kernel for each type of data and then we combine the kernels for a given statistical task such as classification. The simplest combination of kernels is the positive linear combination of them, but other mathematical operations, such as multiplication and exponentiation, produce valid kernels.

Let us start by recalling the main ideas of kernel-based approaches.

Given a sample space $\mathcal {X}$, we say that *k* on $\mathcal {X}$ is a real-valued positive definite kernel on $\mathcal {X}$ if $k:\mathcal {X}\times \mathcal {X}\rightarrow \mathbb {R}$ is a map such that:

- *k*(***x***,***y***)=*k*(***y***,***x***),
- $\sum _{i,j=1}^{m}\alpha _{i}\alpha _{j}k(\boldsymbol {x}_{i},\boldsymbol {x}_{j})\geq 0$ for all $m\in \mathbb {N}$, $\alpha _{i}\in \mathbb {R}$, $\boldsymbol {x}_{i}\in \mathcal {X}$ where *i*=1,…,*m*.

Thus, a kernel can be interpreted as a similarity measure of the samples and allow us to identify each $\boldsymbol {x}\in \mathcal {X}$ with a real function given by

$$\begin{array}{@{}rcl@{}} \boldsymbol{\phi}:\mathcal{X} &\rightarrow & \mathbb{R}^{\mathcal{X}}=\{f:\mathcal{X} \rightarrow \mathbb{R}\}\\ \boldsymbol{x}&\mapsto&\boldsymbol{\phi}(\boldsymbol{x})(\cdot)=k(\cdot,\boldsymbol{x}) \end{array} $$

which is an element of a dot product vector space, from now on referred to as a feature space. It consists of all functions

$$ f(\cdot)=\sum_{i=1}^{m}\alpha_{i}k(\cdot,\boldsymbol{x}_{i}) $$

for any $m\in \mathbb {N}$ and $\boldsymbol {x}_{1},\ldots,\boldsymbol {x}_{m}\in \mathcal {X}$, $\alpha _{1},\ldots,\alpha _{m}\in \mathbb {R}$. It has the reproducing property

$$ \langle k(\cdot,\boldsymbol{x}),f \rangle=f(\boldsymbol{x}) $$

implying 〈***ϕ***(***x***),***ϕ***(***y***)〉=〈*k*(·,***x***),*k*(·,***y***)〉=*k*(***x***,***y***). We can turn our feature space into a Hilbert space $\mathcal {H}_{k}$ by completion. The space $\mathcal {H}_{k}$ is the *reproducing kernel Hilbert space* (RKHS), induced by the kernel function *k*. This remarkable property has important consequences. Indeed, “geometric” intuition can be used to build kernel-based methods, by drawing inspiration from classical statistical methods working in finite dimensional Euclidean spaces. Popular examples of kernel-based methods are kernel principal component analysis (KPCA), kernel ridge regression (KRR), and support vector machines (SVMs) [6, 7].

### Mean element

A natural question to raise is how a probability distribution $\mathbb {P}$ is represented in an RKHS $\mathcal {H}_{k}$. We show that infinite-dimensional counterparts of a fundamental multivariate statistical concept, the mean vector, is particularly appropriate for this purpose. This RKHS-counterpart of the mean vector is known as the mean element.

Consider a random variable *X* taking values in $\mathcal {X}$ and a probability distribution $\mathbb {P}$. The mean element $\mu _{\mathbb {P}}$ associated with *X* is the unique element of the RKHS $\mathcal {H}_{k}$, such that, for all $f\in \mathcal {H}_{k}$

$$\langle\mu_{\mathbb{P}},f\rangle_{\mathcal{H}_{k}}=\mathbb{E}_{\mathbb{P}}[f(X)]. $$

In statistics, a central concern of the data integration outlined above is often referred to as the two-sample or the homogeneity problem. In this study, we explore a test statistic, known as the maximum mean discrepancy (MMD) [8–11], to test whether two samples are from the same distribution. The MMD test can easily be computed using the “kernel trick”. We apply the MMD test to evaluate the differential expression of a set of genes involved in certain metabolic pathways in different conditions. The kernel method allows us integrate metabolomics data with transcriptomic data and so test the homogeneity between conditions, while handling all the available data.

## Methods

Maximum mean discrepancy statistic was designed with the aim to determine a function such that its expectation differs when observations come from two different probability distributions. The underlying premise is that if we compute this statistic on samples drawn from different distributions it will measure how these distributions are likely to differ. This consideration leads to the following statistic. Let $\mathcal {X}$ denote our input domain which is assumed to be a nonempty compact set. Let $\mathcal {F}$ be a class of functions $f:\mathcal {X}\rightarrow \mathcal {F}$. Let $\mathbb {P}$ and $\mathbb {Q}$ be probability distributions, and let *X*=(***x***_1_,…,***x***_*m*_) and *Y*=(***y***_1_,…,***y***_*n*_) be samples composed of independent and identically distributed observations drawn from $\mathbb {P}$ and $\mathbb {Q}$, respectively. The MMD is defined as

$$\text{MMD}[\mathcal{F},\mathbb{P},\mathbb{Q}]=\sup_{f\in\mathcal{F}}\left(\mathbb{E}_{\mathbb{P}}[f(\boldsymbol{x})]-\mathbb{E}_{\mathbb{Q}}[f(\boldsymbol{y})]\right), $$

and its empirical estimate is defined as

$$\text{MMD}[\mathcal{F},X,Y]=\sup_{f\in\mathcal{F}}\left(\frac{1}{m}\sum_{i=1}^{m}f(\boldsymbol{x}_{i})-\frac{1}{n}\sum_{i=1}^{n}f(\boldsymbol{y}_{i})\right). $$

By choosing $\mathcal {F}$ to be the unit ball in a universal RKHS [12] we achieve a desirable tradeoff between a class of functions where $\text {MMD}[\mathcal {F},\mathbb {P},\mathbb {Q}]$ will vanish only if $\mathbb {P}=\mathbb {Q}$ and a class of functions such that the statistic differs significantly from zero for most finite samples *X* and *Y*.

When $\mathcal {F}$ is the unit ball in a universal RKHS, Theorem 2.2 in [8] ensures that $\text {MMD}[\mathcal {F},\mathbb {P},\mathbb {Q}]$ will recognize any discrepancy between $\mathbb {P}$ and $\mathbb {Q}$. Moreover, the finite sample computation of MMD is greatly simplified. Under the assumptions of the aforementioned theorem, the following is an unbiased estimator of $\text {MMD}^{2}[\mathcal {F},\mathbb {P},\mathbb {Q}]$:

$$ {{\begin{aligned} {}\text{MMD}^{2}[\mathcal{F},X,Y]&=\frac{1}{m(m-1)}\sum_{i\neq j}^{m}k(\boldsymbol{x}_{i},\boldsymbol{x}_{j})+\frac{1}{n(n-1)}\sum_{i\neq j}^{n}k(\boldsymbol{y}_{i},\boldsymbol{y}_{j})\\ &\quad-\frac{2}{mn}\sum_{i\neq j}^{m,n}k(\boldsymbol{x}_{i},\boldsymbol{y}_{j}). \end{aligned}}}  $$

A two-sample test based on the asymptotic distribution of an unbiased estimate of MMD^2^ was introduced in [8]. The estimation of the *p*-value of the test can be addressed by sampling. From the aggregated data *Z*={*X,Y*}, we draw randomly without replacement to obtain two new *m*-samples {*X*^∗^,*Y*^∗^}, and compute the test statistic $\text {MMD}^{2}[\mathcal {F},X^{*},Y^{*}]$ on these new samples. If we repeat this procedure *t* times, a set of test statistics under the null hypothesis is obtained:

$${{\begin{aligned} {}\text{MMD}^{2}[\!\mathcal{F},X^{1*},Y^{1*}]\!, \; \text{MMD}^{2}[\!\mathcal{F},X^{2*},\!Y^{2*}]\!,\ldots, \; \text{MMD}^{2}[\!\mathcal{F},X^{t*},\!Y^{t*}] \end{aligned}}} $$

Then, we add the original statistic $\text {MMD}^{2}[\mathcal {F},X,Y]$ to this set, and sort the set in ascending order. Finally, if *r* denotes the position of $MMD^{2}[\mathcal {F},X,Y]$ withing this ordering, the estimation of the p-value is given by $p=\frac {t+1-r}{t+1}$.

We compare the performance of the MMD test with those of other methods, such as the Hotelling test [13]. This is a multivariate generalization of the t-test with a multivariate normal distribution and an identical covariance structure. Alternatively, we also run a multivariate generalization of two well-established model-free univariate tests, the Wald-Wolfowitz runs test and Kolmogorov-Smirnov statistic [14], which is based on the idea of minimum spanning tree (MST). A spanning tree of a graph is a spanning subgraph that is a graph so it provides a path between every two nodes of the graph. Moreover, the MST of an edge-weighted graph is a spanning tree whose edges sum to minimum weight. In the multivariate two-sample problem, it can regard an edge-weighted graph that it is based on the *N* pooled multivariate data in $\mathbb {R}^{p}$ nodes, where *p* is the number of variables of the multivariate data, and edges linking all pairs. The edge weight can be estimate by the Euclidean (or any other) distances between the nodes (pairs of multivariate data). Thus, similar nodes have similar distances. The test is based on the construction of the MST of the pooled multivariate data, then it deletes all edges for which the defining nodes originate from different multivariate samples and, finally, it counts the number of disjoint subtrees (*R*). For large sample sizes, the permutation distribution of the standardized number of subtrees

$$W = \frac{R- E(R)}{\sqrt{var(R)}} $$

approaches the standard normal distribution and the null hypothesis, $\mathbb {P}=\mathbb {Q}$, is rejected for a small number of subtrees [14]. The multivariate Kolmogorov-Smirnov test used the MST to ranking multivariate data. Then, the MST tends to connect nodes (points) that are close. The ranking procedure begins by selecting the root the MST, that is, a node with the largest eccentricity, and then, the nodes are ranked in accordance with the height directed preorder traversal of the tree.

## Results and discussion

To illustrate this procedure, we analyze data from a study in the fields of metabolomics and genomics. Specifically, the datasets are drawn from a nutrigenomic study in the mouse [15]. Forty mice were studied and two sets of variables were acquired from liver cells: 1) expressions of 120 genes derived from a nylon macroarray with radioactive labeling; and 2) concentrations of 21 hepatic fatty acids measured by gas chromatography. Biological units (mice) were cross-classified according to two factors: genotype, in either wild-type (wt) or in PPAR-deficient (ppar) mice; and diet, for which five classes (coc, fish, lin, ref, sun) were identified based on fatty acid composition (Table 1). Specifically, the oils used for experimental diet preparation were corn and colza oils (50/50) for a reference diet (ref), hydrogenated coconut oil for a saturated fatty acid diet (coc), sunflower oil for an Omega6 fatty acid-rich diet (sun), linseed oil for an Omega3-rich diet (lin) and corn/colza/enriched fish oils for the fish diet (43/43/14).

**Table 1:** Nutrimouse studies

	coc	fish	lin	ref	sun
wt	4	4	4	4	4
ppar	4	4	4	4	4

The experimental design is balanced. There are 20 wild type (wt) mice and 20 PPAR-deficient (ppar) mice. Eight mice, four wt and four ppar mice, were fed each diet

For the complete analysis we used a Gaussian kernel and the hyper-parameter was determined by the sigest function of the kernlab R package [16]. The estimation is based upon the 0.1 and 0.9 quantiles of ||***x***−***x***^′^||^2^. Basically, any value in between these two bounds will produce a good hyper-parameter estimation.

We use the GSAR R package [17] to implement the multivariate Kolmogorov-Smirnov test and multivariate Wald-Wolfowitz runs test.

**Fig. 1:**
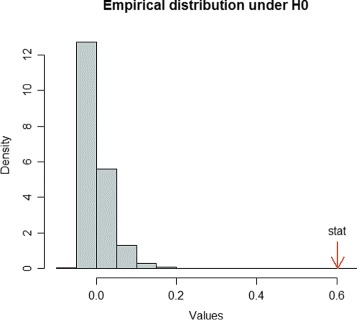
Empirical distribution of kernel MMD under the null hypothesis. The observed value of the test statistic is indicated by an arrow. The number of repetitions is 2499

**Fig. 2:**
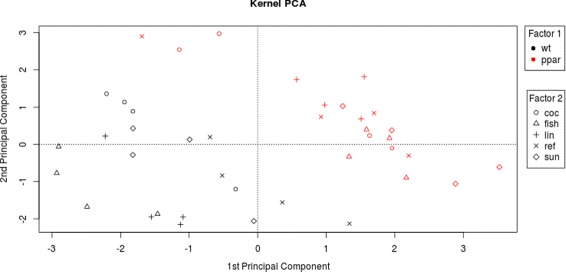
Kernel PCA of gene expression. The wt samples are represented in *black* and the ppar samples in *red*. Diets represented as follows: (ref) diet by *letter x*; (coc) diet by *circles*; (sun) diet by *diamonds*; (lin) diet by *plus signs*; and (fish) diet by *triangles*

**Fig. 3:**
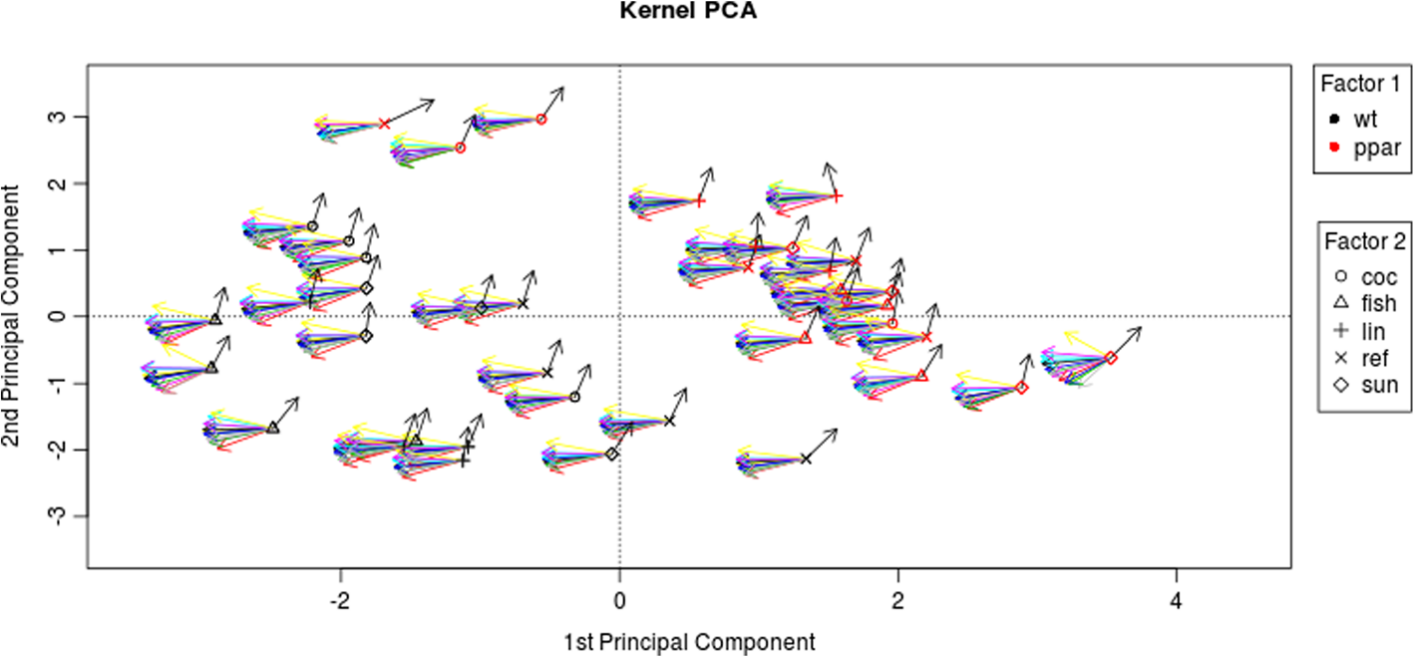
Kernel PCA of gene expression. Kernel PCA of gene expression which shows 16 genes correspond to the fatty acid catabolism pathway. All genes have approximately the same direction (*black vector*) to *left* except ACOTH gene

**Fig. 4:**
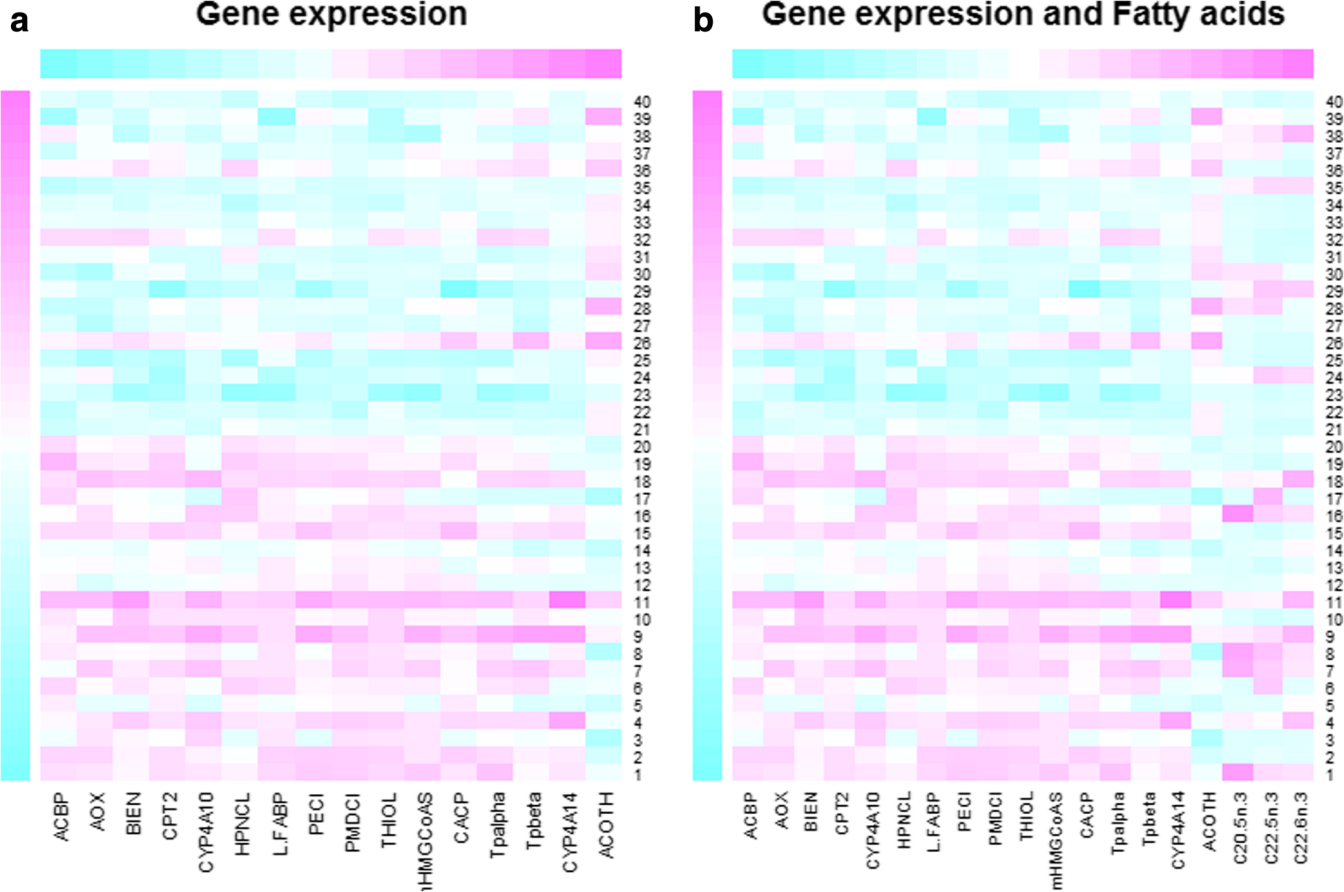
Heatmaps. Figure shows the expression of the fatty acid catabolism pathway between wt and ppar genotype from gene expression (*left*) and gene expression and fatty acids (*right*). Mice from 1 to 20 are wt and from 20 to 40 are ppar

With kernel MMD, we test whether a fatty acid catabolism pathway is differentially expressed in wt vs ppar mice. We consider a set of 16 genes involved in this catabolic pathway: PECI, MDCI, HPNCL, AOX, BIEN, THIOL, CACP, CPT2, TP *α*, TP *β*, mHMGCoAS, Cyp4a10, Cyp4a14, ACBP,L-FABP, ACOTH and PLTP. Using a permutation procedure based on 2499 repetitions, we obtain a significant p-value (Table 2, Fig. 1). Also the three baseline tests are significant (Table 2). The kernel MMD test shows that fatty acid catabolism genes are differentially distributed in wt vs ppar mice. This result, moreover, agrees with the data representation obtained by kernel PCA, which is used to explore simultaneously samples and genes. On the one hand, this projection shows a broad separation between wt and ppar mice (Fig. 2, samples only); on the other, each gene involved in the fatty acid catabolism pathway is displayed as an arrow in each sample (Fig. 3, both samples and pathway genes). Locally, arrows indicate the direction of maximum growth of the gene expression [18]. In Fig. 3, all genes present approximately the same direction to the left, with the exception of the ACOTH gene. Notice that wt mice lie to the left of the first axis and ppar lie to the right (Fig. 2), and by taking into account the direction of the vectors (Fig. 3), we can deduce which genes are overexpressed in wt or, in contrast, in ppar mice. Thus, we can see that the ACOTH gene is the only gene to show a higher expression in ppar mice (Fig. 3). Figure 4 (left) shows a heatmap of this set of genes in which we can observe a pattern of expression that agrees with the interpretation based on the representation of genes using kernel PCA.

**Table 2:** *P*-values in testing fatty acid catabolism pathway

	Genes				Genes and Fatty acids			
	MMD	Hotelling	mKS	mWW	MMD	Hotelling	mKS	mWW
wt vs ppar	4e-04	1e-13	0.001	0.002	0.0096	3e-12	0.002	0.001
sun vs fish	0.1844	-	0.077	0.211	4e-4	-	0.001	0.001

Columns record the MMD, Hotelling, multivariate Kolmogorov-Smirnov (mKS) and multivariate Wald-Wolfowitz runs test (mWW) *p*-values. The first four columns (left) correspond to the pathway representation based on genes, and the second four (right) correspond to the representation based on the integration of genes and fatty acid levels

We exploit the ability of the kernel method to integrate data and so add hepatic fatty acid levels in the pathway to evaluate the test procedure. We consider a set of three fatty acids: C20.5 *ω*.3, C22.5 *ω*.3 and C22.6 *ω*.3 involved in fatty acid catabolism [15]. Thus, we compute the kernel matrix associated with the new feature space (including gene expression and fatty acid levels) by adding the kernel matrix of the gene expression and the kernel matrix of the fatty acid levels. Using a permutation test based on 2499 repetitions, we obtain a significant p-value when testing (Table 2). The heatmap (Fig. 4, right) presents gene expressions in addition to the fatty acid levels, showing that the differences in fatty acids are not so evident between the wt and ppar genotypes. This can be explained by the confounding effect of the diets.

We also studied the effect of the diet on the catabolism pathway. In particular, we compare sun vs fish diets. In this case, the number of samples is less than the number of variables (genes+fatty acids). Kernel methods allow us to avoid this issue in contrast to the classical Hotelling test that does not.

**Fig. 5:**
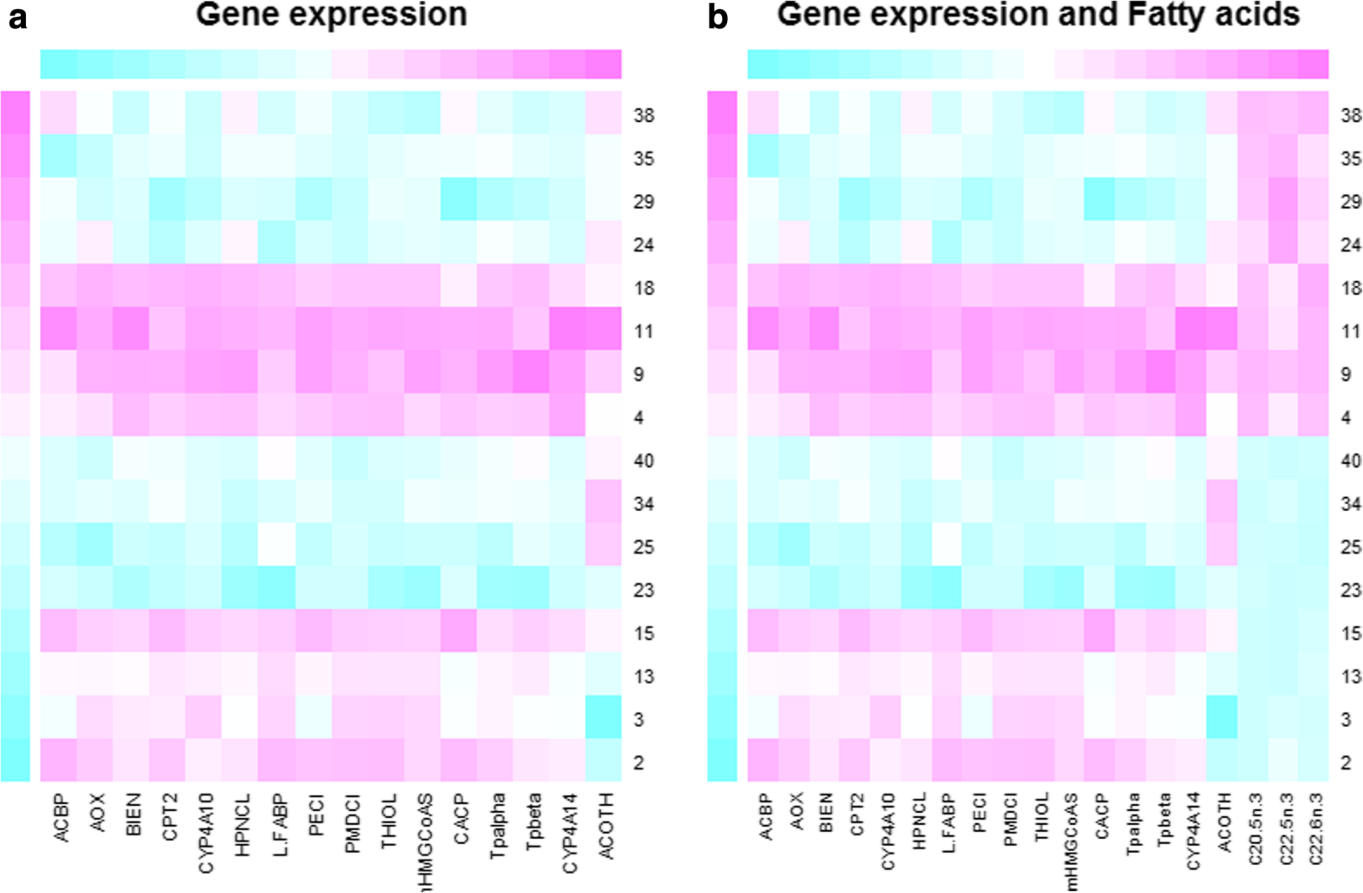
Heatmaps. Figure shows the expression of fatty acid catabolism pathway between sun and fish diet from gene expression (*left*) and gene expression and fatty acids (*right*). Mice 2, 3, 13, 15, 23, 25, 34, 40 were fed the sun diet and the others the fish diet

In addition, the heatmap of the genes (Fig. 5, left) shows an effect of the type of mouse (wt/ppar) but not of the diet. However, when the fatty acid levels are included in the analysis (Fig. 5, right), we observe a different pattern of expression between the diets; that is, the fish diet promotes the levels of this set of fatty acids. Using a permutation test based on 2499 repetitions, we obtain a non-significant *p*-value when the pathway is represented only by the gene expressions. In contrast, when the fatty acid levels are added, the *p*-value is significant. The multivariate Kolmogorov-Smirnov statistic and Wald-Wolfowitz runs test have similar *p*-values (Table 2).

The R source code and example can be consulted at [19] so the experiment can be reproduced.

## Conclusion

MMD is a non-parametric test that acquires several advantages when apply the kernelization of the test: 1) the number of variables can be greater than the sample size; 2) omics data can usefully be integrated; 3) it can be applied not only to vectors, but to strings, sequences and other common structured data types arising in molecular biology. Our results indicate that the kernel MMD can be used to identify differentially expressed pathways; however, further studies with several sets of pathways are needed in order to assess its overall performance. This study suggests that kernel MMD is a useful approach to the analysis of pathway differential expression, since it takes into account all the genes involved in the pathway and, moreover, offers the possibility of integrating several data types.

## Abbreviations

KPCA: kernel principal component analysis
KRR: kernel ridge regression
MMD: maximum mean discrepancy
MST: minimum spanning tree
PCA: principal component analysis
RKHS: reproducing kernel Hilbert space
SVM: support vector machine
